# New hope for the treatment of recurrent and refractory psoriasis: NK cell immunotherapy—A scientometric analysis

**DOI:** 10.3389/fimmu.2025.1656398

**Published:** 2025-10-23

**Authors:** Li Chen, Lin Cheng

**Affiliations:** ^1^ Dermatology Department, Zhuzhou 331 Hospital, Zhuzhou, China; ^2^ Science and Education Department, Zhuzhou 331 Hospital, Zhuzhou, China

**Keywords:** psoriasis, natural killer cells, trained immunity, metabolic reprogramming, immunotherapy, bibliometrics

## Abstract

**Background:**

Psoriasis is a chronic, relapsing, immune-mediated skin disease with a complex pathogenesis and significant individual variability in treatment response. It is characterized by a high relapse rate. In recent years, the critical role of natural killer (NK) cells in the onset and relapse of psoriasis has increasingly attracted attention, and related immune intervention strategies have become a research hotspot.

**Objective:**

This study aims to systematically review the research progress in the field of “NK cells and psoriasis” using bibliometric methods. It identifies research hotspots and key trends, constructs the knowledge structure of this field, and provides data support for related mechanistic studies and clinical translation.

**Methods:**

Based on the Web of Science Core Collection and PubMed databases, we retrieved relevant English-language literature from 2000 to 2025. Using tools such as CiteSpace and VOSviewer, we conducted keyword clustering, author and institutional collaboration analysis, journal co-citation analysis, and highly cited literature mining to create knowledge maps.

**Results:**

A total of 432 publications were included, with a sustained increase in the number of publications over time. The United States, China, and Germany were the main contributing countries, while Rockefeller University and Karolinska Institute were core institutions. The journal Frontiers in Immunology was identified as an important publication venue. Current research focuses on three hotspots: (1) the role of tissue-resident NK cells in “trained immunity” during psoriasis relapse; (2) metabolic reprogramming of NK cells in lesions, with a close correlation between their metabolic status and pro-inflammatory functions; and (3) the development of NK cell-targeted therapeutic strategies, such as dimethyl fumarate (DMF) and IL - 23 inhibitors, which show promising prospects.

**Conclusion:**

This study constructed a comprehensive knowledge map of “NK cells–psoriasis” research, clarifying the hotspots and structural characteristics. Future research will focus on two trends: (1) exploring the pivotal role of NK cells in multi-systemic immune comorbidities and (2) developing specific intervention strategies for different stages of disease progression to advance the development of precision immunotherapy for psoriasis.

## 1 Introduction

Psoriasis is a common chronic immune-mediated skin disease with a strong propensity for recurrence, severely impacting patients’ quality of life. Globally, the prevalence of psoriasis is on the rise, with incidence rates exceeding 2% in some countries and regions (1). Although a variety of biologics and targeted drugs have been developed and achieved certain therapeutic effects in clinical practice, the highly heterogeneous pathogenesis and complex immune regulatory network of psoriasis result in significant individual variability in treatment responses, with a persistently high relapse rate. Achieving clinical remission remains a formidable challenge (2).

In recent years, the pivotal role of the innate immune system in the pathogenesis of psoriasis has been increasingly recognized, particularly the core functions of natural killer (NK) cells in initiating immune responses, amplifying inflammation, and maintaining tissue homeostasis (3). NK cells are involved in the inflammatory processes and relapse reactions of psoriasis through multiple mechanisms, including KIR-MHC recognition, spontaneous release of cytokines (such as IFN-γ and IL - 17), and regulation of the Th17 axis (4, 5). Against this backdrop, immune intervention strategies targeting NK cell functions have garnered increasing attention and have gradually entered the stages of basic research and early clinical validation, offering new insights for addressing the refractory and recurrent nature of psoriasis.

With the development of multi-omics technologies, the functions, spatial localization of NK cells, and their immune network connections with keratinocytes and T cells have been more deeply characterized. However, the field currently lacks a systematic review of the literature, particularly in terms of the overall grasp of literature distribution, research hotspots, core teams, interdisciplinary collaboration, and the application of cutting-edge technologies (6). In this context, the application of bibliometric and visualization analysis techniques can help researchers systematically evaluate the developmental trajectory, research foci, and evolutionary trends of this field. It can also identify key authors, institutions, and collaborative patterns, and provide quantitative decision-making support for future basic and translational research (7).

Therefore, based on the Web of Science Core Collection, this study systematically collected and analyzed English-language literature on “NK cells and psoriasis” from 2000 to 2025. Using bibliometric methods such as keyword clustering, author networks, collaboration maps, and citation analysis, we comprehensively revealed the developmental dynamics, research hotspots, and knowledge structure of this field, aiming to provide theoretical support and a data foundation for further exploring the potential of NK cells in psoriasis treatment.

## 2 Materials and methods

### 2.1 Data source and literature search

The literature data was obtained from the Web of Science Core Collection (WoSCC) database and PubMed database, and the search time was from 1 January 2000 to May 31, 2025. The search formula used was(TS = (psoriasis) AND TS = (“natural killer cell*” OR “NK cell*” OR “CD56+ lymphocyte*” OR “innate lymphoid cell*”).

### 2.2 Data screening

#### 2.2.1 Inclusion criteria

(1) Literature related to natural killer (NK) cells and psoriasis;(2) Literature published in English; (3) Literature types include clinical trial studies, *in vitro* experimental studies, *in vivo* experimental studies, public database analysis studies, reviews, etc.; (4) Literature with complete bibliographic information (including title, country, author, keywords, source).

#### 2.2.2 Exclusion criteria

(1) Conference papers, newspapers, patents, achievements, health and popular science literature, etc.; (2) Duplicate publications;(3) The literature cannot be fully obtained.

The inclusion and exclusion process is independently conducted by two reviewers. If the inclusion and exclusion results are inconsistent, the third reviewer will participate in the work.

#### 2.2.3 Data standardization

After screening, the literature was exported in Refworks and plain text formats. Special characters and redundant spaces were removed. all keywords were standardized by merging synonymous or semantically similar terms into unified categories. For instance, terms such as “2DL1”, “2DS1”, and “killer immunoglobulin-like receptor” were grouped under the unified term “KIR receptors”, reflecting common NK cell recognition molecules. Similarly, variations related to “interleukin” including “IL-17”, “IL-22”, and “IL-23” were unified under the umbrella term “IL cytokines” to highlight key inflammatory mediators. Disease-specific keywords such as “psoriasis arthritis”, “psoriatic lesion”, and “plaque psoriasis” were all merged under “psoriasis” for thematic coherence. Terms referring to immune cell types such as “ILC”, “NK-like cells”, and “innate lymphoid cells” were collectively categorized as “innate lymphoid cells (ILCs)”. Additionally, methodological terms such as “3D imaging”, “mass spectrometry”, and “single-cell RNA-seq” were respectively standardized as “imaging techniques” and “omics technologies”. This standardization process ensured that frequency and co-occurrence analyses accurately reflected research hotspots without terminological redundancy. Country/Region names were standardized for consistency in bibliometric analysis. For example, “Hong Kong”, “Macau”, and “Taiwan” were categorized under “China”, while “Scotland”, “Wales”, and “England” were grouped under “United Kingdom”. Subsequently, the Data Import/Export function in CiteSpace software was used to convert and process the retrieved literature, ensuring the uniformity of metadata for further analysis.

#### 2.2.4 Keyword analysis

The keyword co-occurrence network was constructed by selecting the top N nodes (N = 30) and applying Pruning: Pathfinder + Pruning sliced networks to reduce noise. The thematic density (Density) and centrality (Centrality) were calculated to identify the key nodes in this field. Keyword burst detection was employed to reveal shifts in research hotspots. A temporal evolution map of keywords was generated to illustrate the dynamics of hotspot migration. Furthermore, high-frequency keywords related to NK cells and psoriasis were extracted and subjected to LLR clustering analysis, enabling the identification of major research domains.

#### 2.2.5 Data analysis

##### 2.2.5.1 Data extraction

The normalized text data will be incorporated into a structured form designed by two researchers, who will then extract relevant data. The extracted data includes the following parts: publication information, encompassing the year of publication, country/region, issuing organization, issuing journal, authors, cited literature, and keywords.

##### 2.2.5.2 Analysis methods

**Figure 1:**
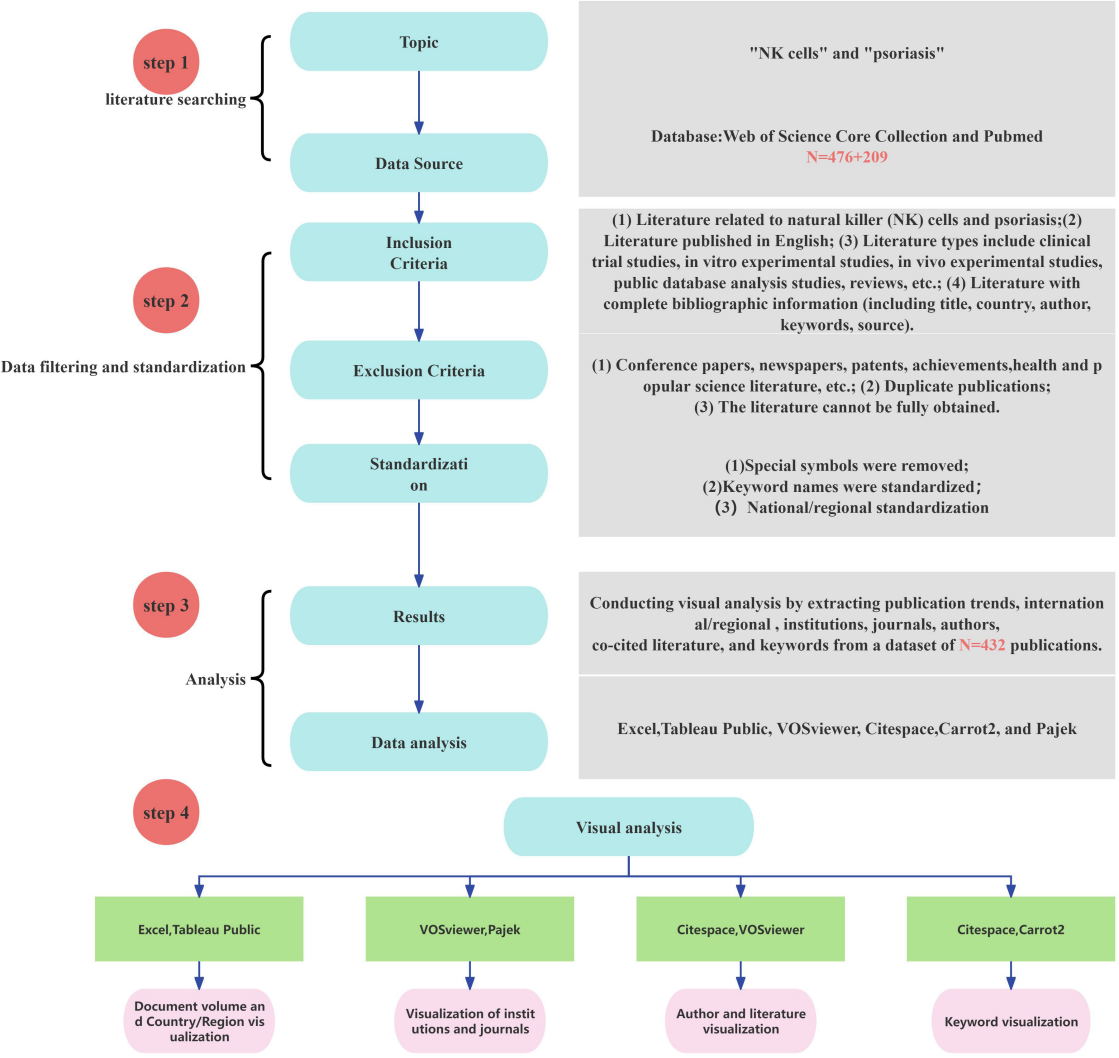
Workflow of scientometric analysis of NK cell immunotherapy in psoriasis research.

In this study, we employed bibliometric visualization analysis to comprehensively organize and uncover valuable hidden information in the field. The deduplication and merging of TXT files retrieved from the Web of Science Core Collection (WOS) and PubMed databases were carried out using Endnote. For the visualization of publication output, the textual data were converted to Excel via Citespace’s data processing tool, and a fitting curve was further plotted to accurately predict future publication trends. The visualization analysis of countries/regions was realized by converting the textual data to Excel using VOSviewer and subsequently visualizing it with Tableau Public. The visualization of publishing institutions was jointly achieved using VOSviewer and Pajek. The data analysis of journals and authors was conducted by exporting the textual data to CSV files via VOSviewer and then organizing them. The keyword visualization analysis involved converting the textual data to XML files using Citespace and subsequently generating bubble charts with Carrot 2. The process of data acquisition and analysis is illustrated in **Figure 1**.

## 3 Results

### 3.1 Publication output

**Figure 2:**
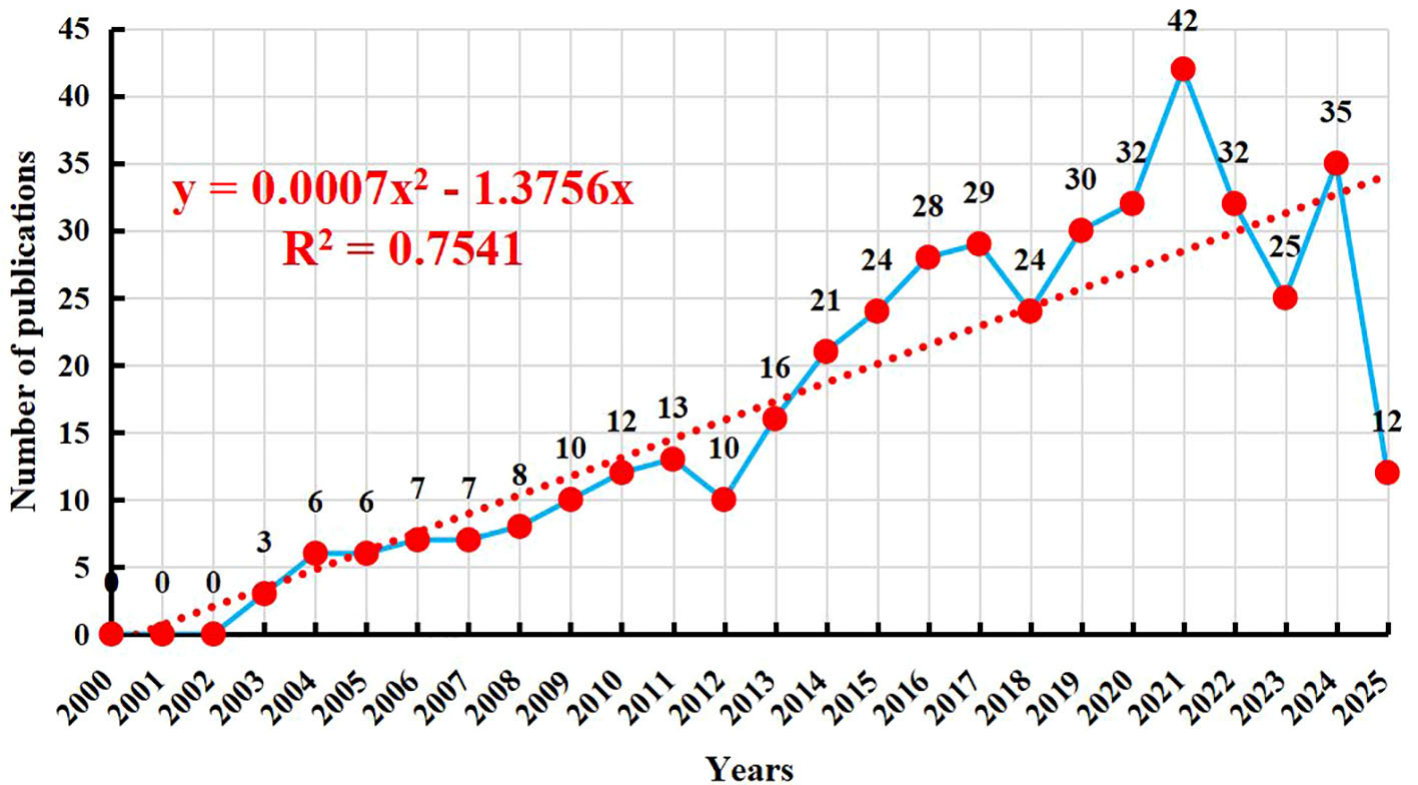
Research trends of NK cell immunotherapy in psoriasis.

**Figure 2** presents the publication trends over the past 20 years. A total of 432 publications were identified in this period. No relevant studies were published between 2000 and 2002. The first sporadic publications appeared in 2003, and the number of publications gradually increased thereafter. From 2003 to 2010, the number of publications slowly rose from 3 to 12. The growth rate accelerated between 2011 and 2015, with the annual publication output increasing from 13 to 24. The period from 2016 to 2021 witnessed a sustained increase, with the annual publication count rising from 28 to a peak of 42, indicating a significant surge in research interest in “NK cells and psoriasis.” There was a slight decline in 2022 (32 publications) and a further decrease in 2023 to 25 publications. However, the number rebounded to 35 in 2024, suggesting that this research direction remains highly relevant. As of the search date in 2025, 12 related publications had already been released, and it is anticipated that the annual publication output will remain at a considerable level for the entire year. Overall, the field has demonstrated a consistent upward trend over the past two decades, particularly in the last decade, which has been a period of active research. To more accurately predict future trends, a polynomial fitting curve was plotted, as indicated by the red dashed line in **Figure 2**. This curve suggests that publications in this field will continue to grow in the future. The coefficient of determination (R² = 0.7541) indicates that the model can explain 75.41% of the data variability, suggesting that this trend is highly informative. The increasing publication output highlights that NK cell immunotherapy in psoriasis is currently a focal point of research and holds promising prospects for future development.

### 3.2 Countries/Regions

**Figure 3:**
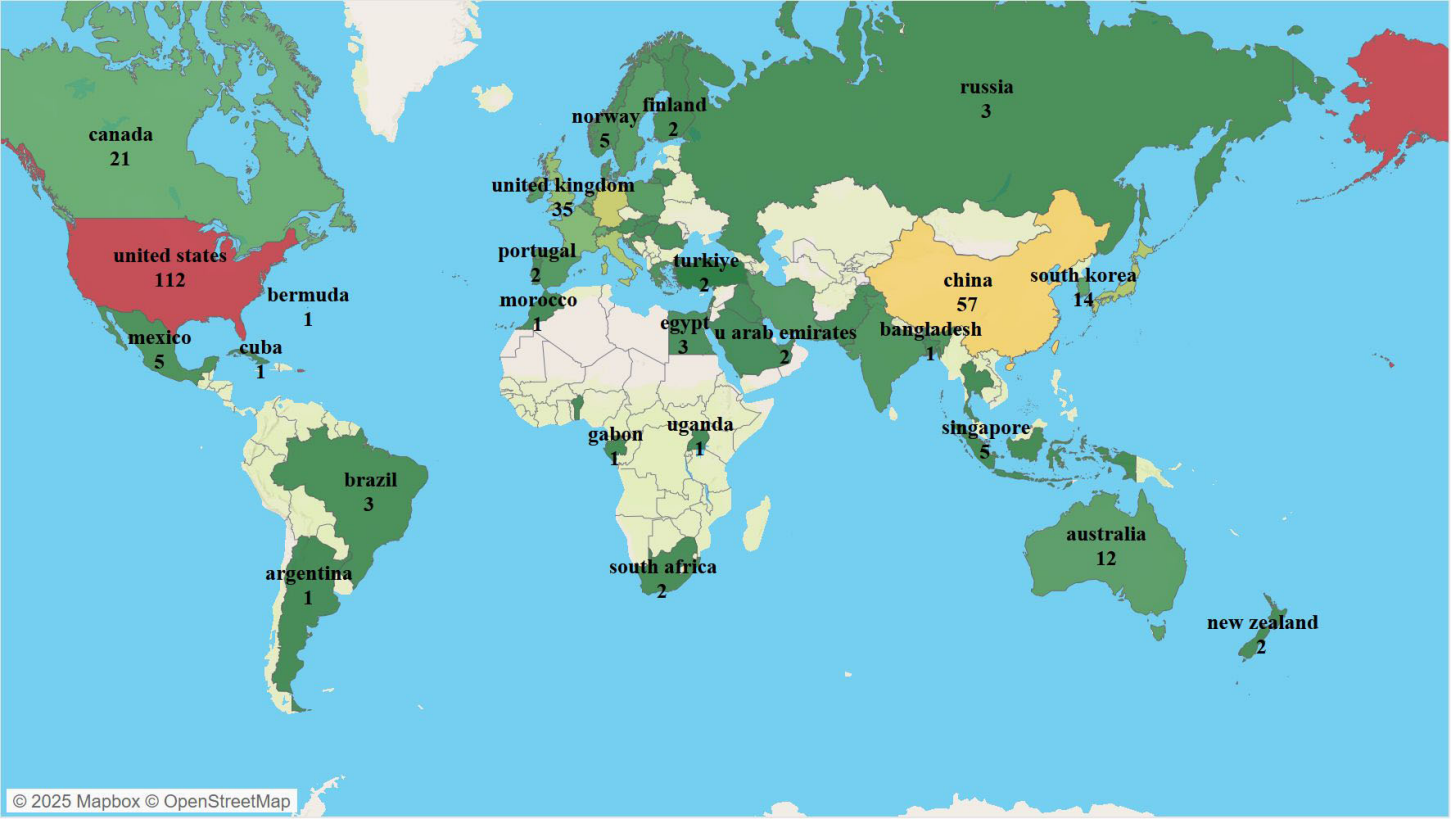
Visualization of Countries/Regions in NK Cell Therapy Research for Psoriasis; colors and numbers represent the number of publications from each country in this field.

Over the past 20 years, scholars from 60 countries/regions worldwide have contributed to publications related to NK cells and psoriasis. As shown in **Figure 3**, the geographical distribution of publications is highly concentrated, primarily in developed countries in Europe, America, and East Asia. The United States has the highest number of publications (n = 112), followed by China (n = 57), Germany (n = 49), Japan (n = 43), Italy (n = 42), and the United Kingdom (n = 35). In terms of international collaboration, the United States holds a dominant position (cooperation intensity = 98), followed by Germany (n = 50) and the United Kingdom (n = 41), indicating that European and American countries have established stable collaborative research networks in this field.

**Table 1** provides a detailed overview of the top 10 countries in terms of publication output, cooperation intensity, total citations, and average citations per publication. The United States not only has the highest total citations (n = 12,499) but also the highest average citations per publication (162.22), demonstrating its absolute advantage in producing high-impact research outcomes. Although China ranks second in publication output, its average citation count is 39.09, significantly lower than that of Germany (55.76), the United Kingdom (58.61), and Italy (51.73), indicating that there is room for improvement in research impact.

**Table 1:** The top 10 countries according to the total publications.

Rank	Country	Publication	Cooperation intensity	Total citations	Average citation
1	united states	112	98	12499	162.2241
2	china	57	17	1704	39.0935
3	germany	49	50	5005	55.7616
4	japan	43	12	2521	51.3534
5	italy	42	20	3239	51.7301
6	united kingdom	35	41	4728	58.6111
7	france	30	28	2433	35.139
8	canada	21	20	1733	27.2499
9	netherlands	20	26	2574	30.858
10	switzerland	19	18	1497	22.4632

In summary, research on NK cells and psoriasis has formed a global pattern, with the United States and European countries as the academic core. While Asian countries have shown rapid growth in publication numbers, they still need to enhance international collaboration and accumulate high-quality research outcomes to strengthen their academic impact.

### 3.3 Institution and author

**Figure 4:**
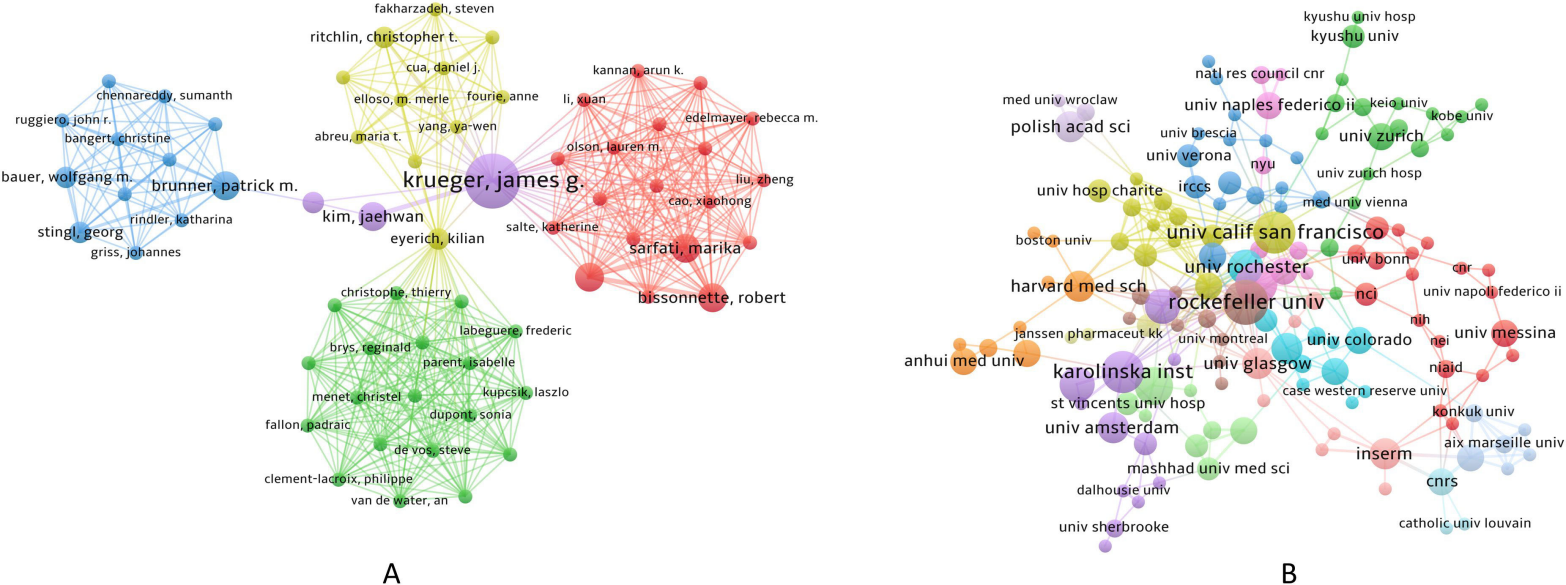
Visualization of Authors and Institutions; **(A)** Author collaboration network; **(B)** Institutional collaboration network; In panel **(A)** the circle size represents the publication frequency of each author, with larger circles indicating higher output; in panel **(B)** the circle size represents the number of publications from each institution, with larger circles indicating higher output.

Over the past 20 years, a total of 2,558 authors from 806 institutions worldwide have published their research findings on NK cells in the field of psoriasis. **Figure 4A** presents the author collaboration network, in which Krueger, James G. (N = 8) is the most prolific author, while Sabat, Robert (C = 1,452) is the most cited author. During this period, five key teams centered around Krueger, James G., Bissonnette, Robert, Eyerich, Kilian, Brunner, Patrick M., and Christophe, Thierry have collectively propelled the development of this field, as shown in **Figure 4B**. **Figure 4C** illustrates the institutional collaboration network, with Rockefeller Univ (N = 10), Karolinska Inst (N = 9), Univ Calif San Francisco (N = 9), Univ Oxford (N = 9), and Trinity Coll Dublin (N = 8) being the most productive institutions in terms of publication output. The Univ Oxford (C = 1,983) is the most cited institution, and Univ Calif San Francisco and Univ Oxford have the highest cooperation intensity. The collective efforts of these authors and institutions have driven the rapid development of this field. The specific information of the authors and institutions with major contributions is shown in **Table 2**.

**Table 2:** Top 10 Contributing authors and institutions.

Author	Number of documents issued	Cited quantity	Cooperation intensity	Institution	Number of documents issued	Cited quantity	Cooperation intensity
krueger, james g.	8	990	29	rockefeller univ	10	1559	19
kastelan, marija	6	209	35	karolinska inst	9	683	16
liao, wilson	5	129	20	univ calif san francisco	9	357	20
sabat, robert	4	1452	20	univ oxford	9	1983	20
spits, hergen	4	633	28	trinity coll dublin	8	597	9
wolk, kerstin	3	1434	20	hannover med sch	7	307	3
witte, ellen	3	991	16	univ manchester	7	866	9
sterry, wolfram	3	828	12	univ rochester	7	110	13
brunner, patrick m.	3	530	11	harvard med sch	6	885	10
kim, jaehwan	3	467	9	inserm	6	245	14

### 3.4 Journals

**Figure 5:**
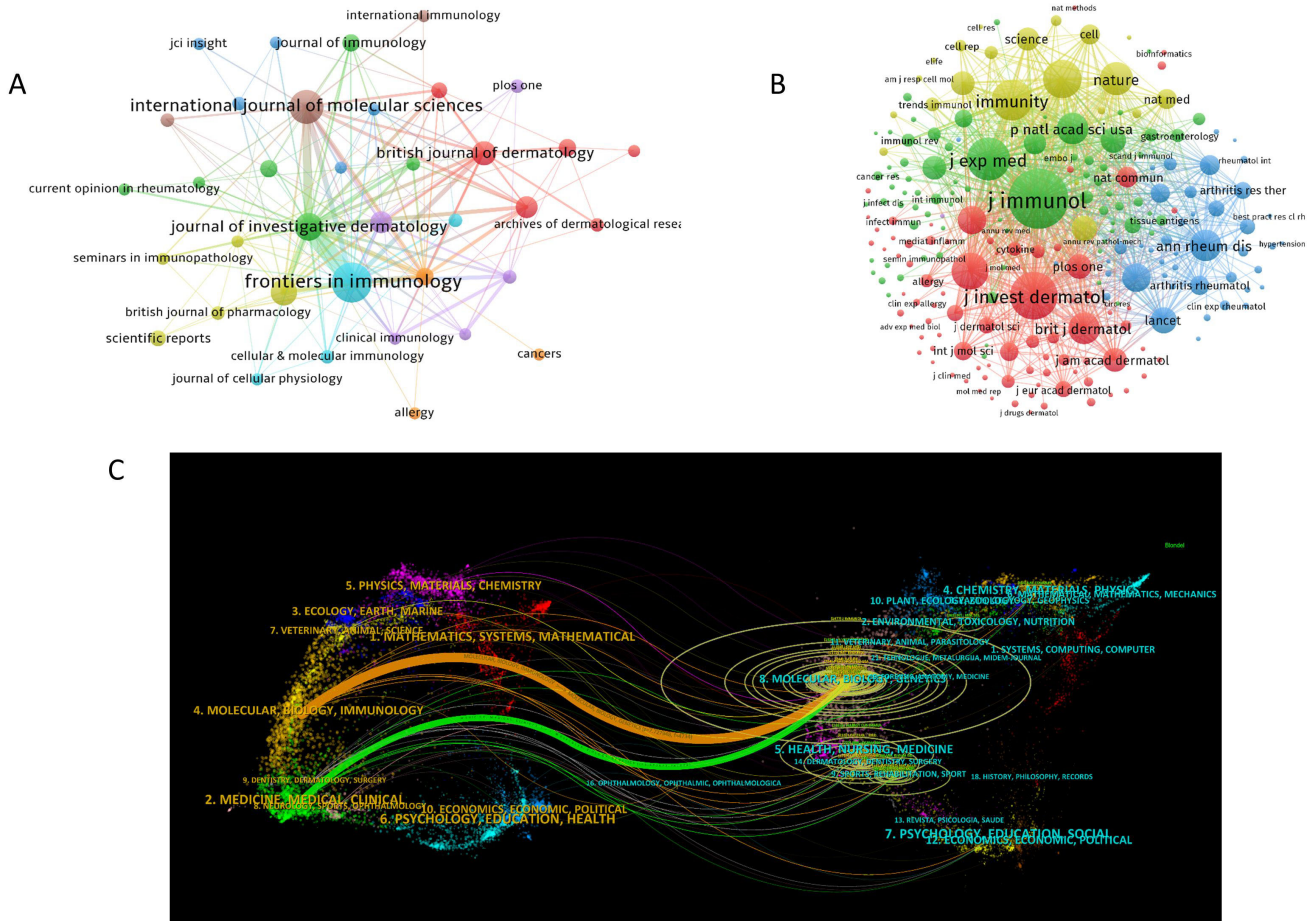
Visualization of Journals in NK Cell Immunotherapy Research for Psoriasis; **(A)**. Network of publication output for journals with citation counts ≥ 3;**(B)**. Co-citation network of journals; **(C)**. Dual-map overlay of journals; In panel **(A)** the circle size represents the citation frequency of journals, with larger circles indicating higher citations; in panel **(B)** the circle size represents the co-citation frequency of journals, with larger circles indicating higher co-citations; in panel **(C)** the left side shows clusters of citing journals and the right side shows clusters of cited journals, with ring size representing citation frequency, while orange and green curves indicate citation paths.

A total of 217 journals have published research findings on NK cell immunotherapy in psoriasis. **Figure 5A** displays journals with citation counts greater than or equal to 3. Among them, Frontiers in Immunology (N = 32), International Journal of Molecular Sciences (N = 23), Journal of Investigative Dermatology (N = 16), Journal of Allergy and Clinical Immunology (N = 14), and British Journal of Dermatology (N = 12) are the most prolific journals in terms of publication output. The journals with the highest citation counts are Frontiers in Immunology (C = 2,151), Nature (C = 1,508), Journal of Allergy and Clinical Immunology (C = 1,419), European Journal of Immunology (C = 1,253), and International Journal of Molecular Sciences (C = 1,182). **Figure 5B** presents the co-citation network of journals, with J. Immunol (CC = 2,479), J. Invest. Dermatol. (CC = 1,620), J. Exp. Med. (CC = 1,359), Immunity (CC = 1,247), and Nat. Immunol. (CC = 1,112) being the most co-cited journals. The dual-map overlay of journals (**Figure 5C**) illustrates the citation relationships between citing and cited journals. The left side represents the clustering of citing journals, indicating the distribution of knowledge frontiers in the field, while the right side represents the clustering of cited journals, highlighting the important knowledge bases of the field. The orange path in **Figure 5C** indicates that journals from the fields of Molecular, Biology, and Genetics are most likely to be cited by journals related to Molecular, Biology, and Immunology, suggesting that the latest research involves substantial interdisciplinary studies. The green path indicates that journals from Molecular, Biology, and Genetics are most likely to be cited by journals related to Medicine, Medicinal, and Clinical, highlighting the multidisciplinary integration and convergence in this field.

### 3.5 Keywords

**Figure 6:**
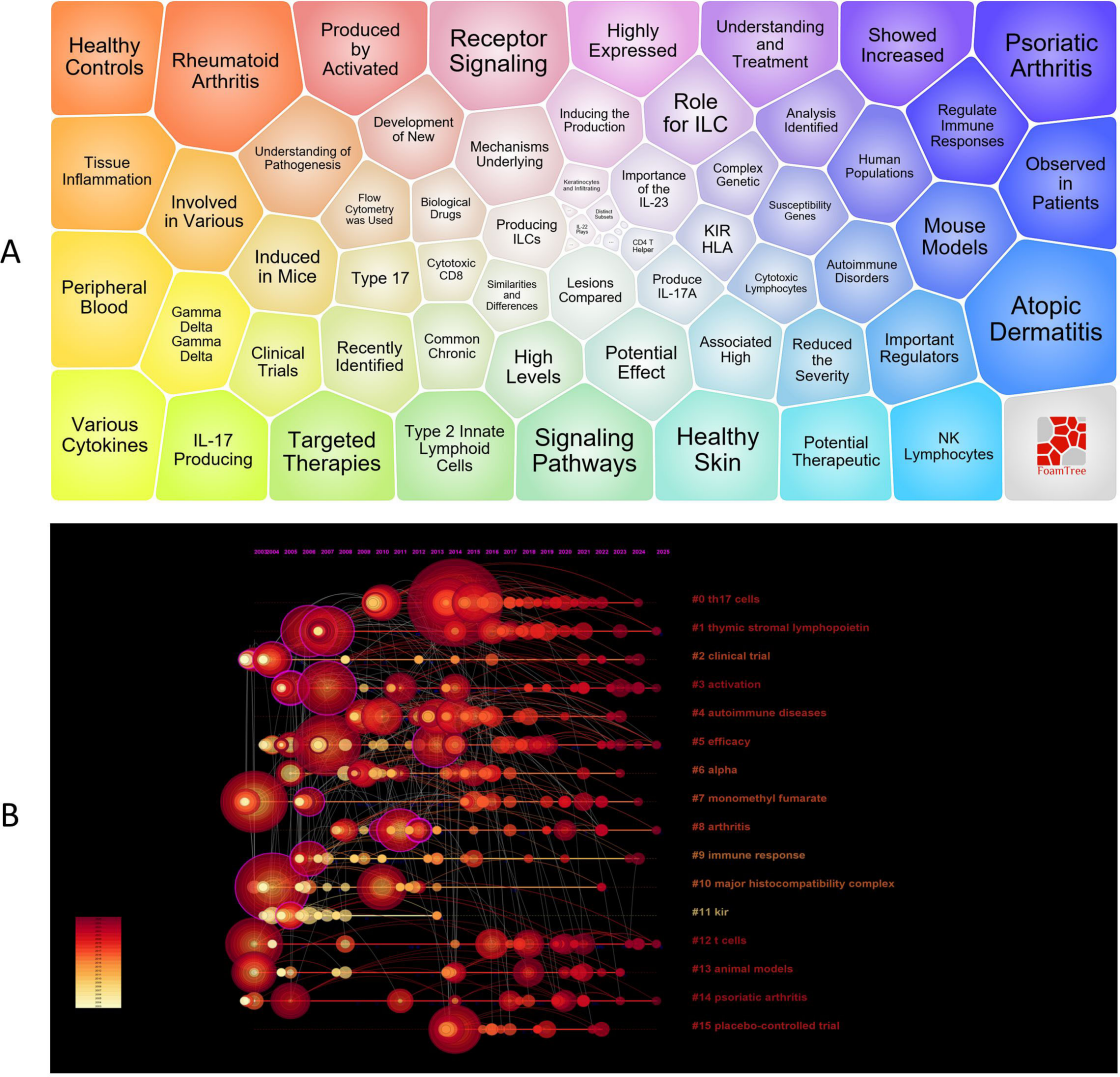
Visualization of Keywords; **(A)** Keyword bubble chart analysis; **(B)** Timeline of keyword clusters; In panel **(A)** larger bubbles represent higher occurrence frequency of keywords. In panel **(B)** the circle size indicates the frequency of keyword occurrence, with larger circles denoting higher frequency. A greater number of circles within the same cluster reflects a higher concentration of research in that area.

Through the visualization analysis of keywords, we obtained key information in the current field. **Figure 6A** presents a bubble chart of keywords, with modules such as inflammation, atopic dermatitis, psoriatic arthritis, autoimmunity, skin, natural killer cells, innate lymphoid cells, IL - 17, innate immunity, and cytokines being the focal points of research. To further analyze the developmental trends of this field over time, **Figure 6B** shows a timeline of key clusters, indicating that 16 main clusters were formed in the field of NK cell immunotherapy and psoriasis research between 2018 and 2025.

Through keyword clustering analysis, we identified three key themes in the field of NK cell immunotherapy and psoriasis research. These themes not only cover the main threads of basic research and clinical translation but also reflect the research progress in immune regulation mechanisms and therapeutic target exploration.

Firstly, the autoimmune pathogenesis of psoriasis (core keywords: #4 autoimmune diseases, #9 immune response, #12 T cells, #14 psoriatic arthritis, #13 animal models) forms the theoretical foundation of the research. As a typical immune-mediated inflammatory disease, psoriasis is closely related to the dysfunction of the adaptive immune system, especially the abnormal activation of the Th17/IL-23 axis mediated by T cells. In-depth studies of T cell functions, IL - 17 production, and keratinocyte responses in animal models have provided an important basis for elucidating the regulatory role of NK cells in the immune network.

Secondly, the activation mechanisms of NK cells and innate immune signaling pathways (core keywords: #1 thymic stromal lymphopoietin, #3 activation, #10 major histocompatibility complex, #11 KIR, #0 Th17 cells) are important research directions in this field. Studies have shown that NK cells not only recognize target cells through KIR-MHC interactions but are also activated by cytokines such as TSLP and IL - 12, inducing Th17 responses and releasing IFN-γ, thereby amplifying and maintaining psoriatic inflammation. These studies provide theoretical support for revealing the cross-regulation mechanisms between innate and adaptive immunity.

Lastly, preclinical and clinical explorations of NK cell-targeted immunotherapy (core keywords: #2 clinical trial, #5 efficacy, #6 alpha, #7 monomethyl fumarate, #15 placebo-controlled trial, #8 arthritis) constitute the translational frontier of this research. In recent years, small molecule drugs (such as monomethyl fumarate) and biologics (such as IL - 17/IL-23 monoclonal antibodies) targeting NK cell functions have shown promising efficacy and safety in improving skin and joint lesions in psoriasis and psoriatic arthritis through multiple randomized controlled clinical trials, laying the foundation for the development of personalized immune intervention strategies.

In summary, the three key themes—from the basic research of psoriasis immune mechanisms to the inflammation amplification pathways mediated by NK cells and the clinical evaluation of immunotherapy—constitute the core knowledge framework of the field of “NK cell immunotherapy and psoriasis,” reflecting a systematic developmental trend from mechanism elucidation to clinical application.

### References

3.6

**Figure 7:**
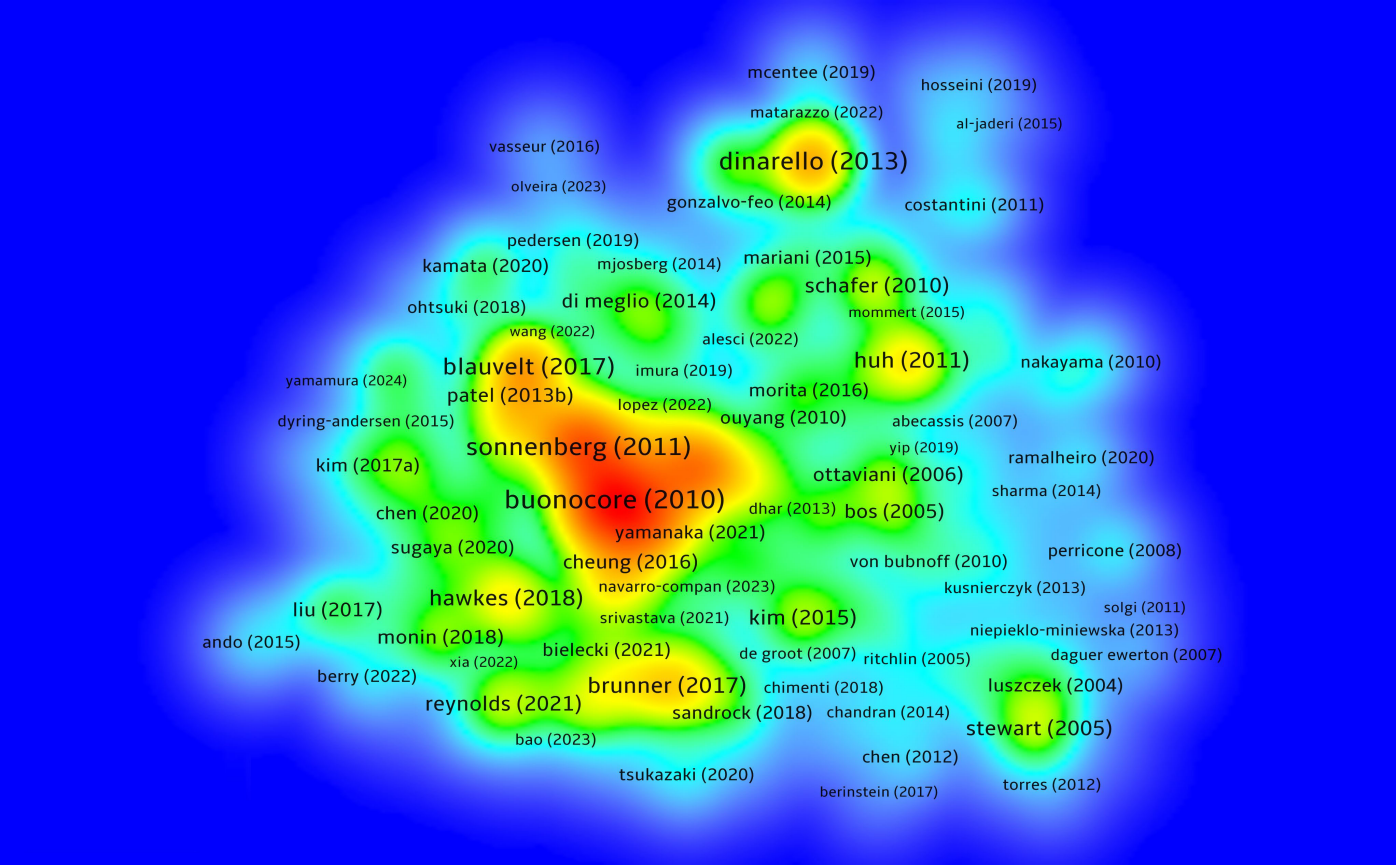
Density map analysis of highly cited articles; The central orange area represents a high-density region, indicating that the literature in this area has been cited more frequently.

By conducting an in-depth analysis of highly cited literature, we can further explore the developmental trends and key turning points of NK cell immune regulation in psoriasis research. **Figure 7** presents a density map of articles cited more than 10 times, highlighting the important positions of some core articles in the co-citation network.

Buonocore et al. (2010) published a study in Nature (8) that first revealed that RORγt+, IL - 23R+ innate lymphoid cells (ILCs) can independently induce IL - 17 and IFN-γ expression in a T cell-deficient Rag– mouse model, driving the occurrence of intestinal inflammation in IL - 23-mediated inflammatory responses. This study clarified the pathogenic potential of ILCs in tissue-specific inflammation and provided a solid experimental basis for IL - 23 pathway-related autoimmune diseases such as psoriasis, occupying a core node in the co-citation network.

Gregory F. Sonnenberg et al. (2011) (9) systematically reviewed the functions of IL - 22 and its receptor pathway in immune regulation of barrier tissues, pointing out that IL - 22 plays a key role in maintaining immune homeostasis, antimicrobial defense, and tissue repair in the skin, gut, and respiratory tract. The study emphasized that the imbalance of IL - 22 expression is closely related to diseases such as psoriasis and inflammatory bowel disease, suggesting that the ILC family (including NK-like cells) participates in chronic inflammation and tissue homeostasis imbalance through the IL - 22 signaling axis. Their theoretical contributions are of great significance for NK cell function research and the development of targeted therapies.

Brunner et al. (2017) (10) reviewed the immune landscape of atopic dermatitis in the Journal of Allergy and Clinical Immunology, emphasizing the central role of ILC2 in TH2-dominated inflammatory responses. The article pointed out that AD is not only characterized by TH2 skewing but also involves TH17, TH22, and partial TH1 responses. ILC2 is significantly enriched in lesions, releasing cytokines such as IL - 5 and IL - 13, leading to barrier disruption and persistent inflammation. This review clearly supports the effectiveness of targeting the IL - 4/IL-13 pathway (e.g., dupilumab) and provides theoretical support for understanding the role of innate immune cells in chronic skin inflammation, including psoriasis.

Villani et al. (2018) (11) constructed a high-resolution atlas of human peripheral immune cells through single-cell RNA sequencing, identifying multiple functionally heterogeneous innate immune cell subsets, including different types of dendritic cells, monocytes, and NK cells. The study revealed the dynamic expression characteristics of ILC family cells in different inflammatory microenvironments, providing foundational resources for further exploring the functional differentiation and pathogenesis of NK cells in complex immune diseases such as psoriasis.

Teunissen et al. (2021) (12) used single-cell multi-omics strategies to analyze the immune features of memory T cells (TRM) and innate lymphoid cells (including ILCs and NK-like cells) in human skin tissue. The study found that psoriatic lesions contain a large number of CD49a+CD103+ NK-like cells that rapidly produce IL - 17 and IFN-γ in response to IL - 23, driving typical Th17-type inflammatory responses. This study proposed that “innate memory” cells may play a central role in psoriasis relapse mechanisms, offering new strategies for targeting TRM and ILCs to intervene in psoriasis relapse. The main findings of the top 20 highly cited articles are further summarized in **Table 3**.

**Table 3:** Milestone findings of highly cited articles (Top 20).

No.	Authors	Citations	Journal	Year	Main Findings	Reference
1	Buonocore S, Ahern PP, Uhlig HH, et al.	914	Nature	2010	Identified a RORγt+ Thy1hiSCA-1+ ILC subset that produces IL - 17 and IFN-γ under IL - 23 stimulation and mediates innate immune colitis in mouse models. Suggests ILCs share functional features with Th17 cells in IL - 23-driven inflammation and may be therapeutic targets in diseases like psoriasis.	(8)
2	Sonnenberg GF, Fouser LA, Artis D	831	Nature Immunology	2011	Systematic review highlighting the key role of IL - 22 and its receptor in immune homeostasis, antimicrobial defense, and tissue repair in barrier tissues. Dysregulated IL - 22 is associated with psoriasis, indicating potential ILC/NK cell involvement via the IL - 22 signaling axis.	(9)
3	Wolk K, Witte E, Witte K, et al.	780	European Journal of Immunology	2006	IL-22 regulates keratinocyte expression of antimicrobial peptides and inflammatory mediators, enhancing innate immunity. Upregulated IL - 22 in psoriasis lesions suggests a major role for IL - 22 in NK cell-mediated pathogenesis.	(13)
4	Ouyang W, Rutz S, Crellin NK, Valdez PA, Hymowitz SG	766	Frontiers in Immunology	2011	Review on IL - 22 biology and dual roles in tissue protection and immunopathology. Highlights IL - 22 from NK cells and ILC3 in psoriasis pathogenesis via keratinocyte proliferation and inflammation amplification.	(14)
5	Villanova F, Flutter B, Tosi I, et al.	667	Journal of the American Academy of Dermatology	2017	Confirmed the presence of IL - 17-producing ILC subsets (including NK cells and ILC3) in psoriasis lesions, indicating T cell-independent inflammation and diverse cellular sources downstream of IL - 23–IL-17 axis.	(15)
6	Arakawa A, Siewert K, Stohr J, et al.	503	Clinical Reviews in Allergy & Immunology	2018	Reviewed psoriasis-related immune networks, stressing the complexity of IL - 17-producing cells including NK cells and ILC3, supporting therapies targeting non-T cell-derived IL - 17.	(16)
7	Cochez PM, Michiels C, Hendrickx E, et al.	461	Therapeutic Advances in Chronic Disease	2019	Reviewed IL - 23/IL-17 axis and its effector cells in psoriasis, highlighting NK/ILC3 synergism in IL - 17-mediated immunity and non-T cell inflammation.	(17)
8	Wolk K, Kunz S, Asadullah K, Sabat R	460	Journal of Immunology	2004	IL-22 acts on keratinocytes to induce antimicrobial and inflammatory factors, promoting proliferation and inflammation. First proposed pathogenic role of IL - 22 in chronic skin diseases like psoriasis.	(18)
9	Brunner PM, Guttman-Yassky E, Leung DYM	458	Journal of Allergy and Clinical Immunology	2017	Outlined immune landscape of atopic dermatitis, emphasizing ILC2 role and TH17/TH22/TH1 co-activation. Provides insight into innate immunity in psoriasis and related skin diseases.	(10)
10	Boyman et al.	421	J Exp Med	2004	Established a new model of human pre-psoriatic skin forming psoriasis-like lesions in AGR129 mice, showing crucial roles of resident T cells and TNF-α.	(19)
11	Simoni Y, Fehlings M, Kløverpris HN, et al.	418	Immunity	2018	Created a single-cell RNA-seq atlas of human ILCs and NK cells in peripheral and tissue compartments. Identified skin-resident NK cells expressing CD49a/CD103, suggesting pathogenic potential in inflammatory skin diseases like psoriasis.	(20)
12	Teunissen MBM, Munneke JM, Bernink JH, et al.	398	Immunity	2021	Single-cell analysis revealed enrichment of CD49a+CD103+ NK-like cells in psoriatic lesions. These cells release IL - 17 and IFN-γ under IL - 23 stimulation, indicating a key role in psoriasis recurrence and chronic inflammation.	(21)
13	Stewart CA, Laugier-Anfossi F, Vély F, et al.	322	Nature Immunology	2005	First demonstrated that activating KIRs recognize peptide-MHC-I complexes, advancing understanding of NK receptor-mediated self-recognition and activation relevant to immune diseases like psoriasis.	(22)
14	Schafer PH, Parton A, Capone L, et al.	321	British Journal of Pharmacology	2010	Apremilast, a PDE4 inhibitor, suppresses IL - 12, IL - 23, and TNF-α production, showing strong anti-inflammatory effects *in vitro* and *in vivo*. Suggests indirect modulation of innate immune cells including NK cells.	(23)
15	Liew PX, Kubes P	311	Nature Reviews Immunology	2019	Reviewed the lineage, activation, and regulatory roles of tissue-resident innate immune cells including NK and ILCs. Emphasized their non-redundant roles in peripheral tissue inflammation such as in psoriasis.	(24)
16	Villanova F, Flutter B, Tosi I, et al.	301	Journal of Investigative Dermatology	2014	Identified CD56+CD161+ NK-like cells in psoriatic lesions producing IL - 17 upon IL - 23 stimulation without antigen presentation, highlighting their importance in Th17-like inflammation amplification.	(25)
17	Spits H, Artis D, Colonna M, et al.	289	Nature Reviews Immunology	2013	Comprehensively reviewed ILC development, phenotype, and function. Stated that ILC3 and NK cells play complementary and synergistic roles in inflammatory skin diseases like psoriasis.	(26)
18	Gronke K, Kofoed-Branzk M, Textor S, et al.	282	Immunity	2017	Showed IL - 22 can be produced by NK and NKT cells as well as ILC3s. Mouse models confirmed IL - 22+ NK cells regulate inflammation in gut and skin, supporting a multifunctional role in psoriasis.	(27)
19	Gálvez J	274	Immunologic Research	2014	Reviewed crosstalk between IL - 23/IL-17 axis and innate lymphoid cells in gut and skin inflammation. Emphasized ILCs, including NK cells, as key IL - 17 sources in chronic inflammatory diseases like psoriasis.	(28)
20	Glennie ND, Volk SW, Radtke AJ, et al.	263	Nature Immunology	2015	Demonstrated that skin-resident memory-like NK cells can form independently of T cells via IL - 15 and mount enhanced IFN-γ responses upon secondary stimulation, suggesting a role in psoriasis relapse.	(29)

## 4 Discussion

### 4.1 Research hotspots

#### 4.1.1 *In vivo* studies: the role of NK cells in psoriasis relapse

Recent studies have consistently highlighted the central immunological functions of NK cells in psoriasis relapse (30). Beyond their early role in disease onset by secreting IL - 17 and IFN-γ to drive inflammation, specific subsets of tissue-resident NK cells (trNK) exhibit “trained immunity” features. These cells can persist in the skin during remission and, upon re-exposure to inflammatory cytokines such as IL - 23 and IL - 15, are rapidly reactivated to reinitiate Th17-like inflammatory pathways (30).

Another study using a chronic skin inflammation model emphasized that trNK cells display strong chemotaxis and metabolic adaptability, forming a positive feedback inflammatory network with keratinocytes and dermal dendritic cells, thereby sustaining chronic lesions (3). Since their activation is independent of antigen presentation, trNK cells are often refractory to conventional T cell–targeted therapies, suggesting that they may serve as “hidden drivers” of disease recurrence.

#### 4.1.2 *In vitro* studies: functional phenotypes and inflammatory mechanisms

*In vitro* experiments simulating the psoriatic microenvironment have shown that NK cells significantly upregulate pro-inflammatory cytokine expression and activate STAT3 and NF-κB pathways under IL - 23 and IL - 15 stimulation. This pro-inflammatory phenotype, when co-cultured with keratinocytes, creates an amplified inflammatory loop, underscoring their potential role in sustaining psoriasis inflammation. Evidence further indicates that CD49a^+^CD103^+^ NK-like cells enriched in psoriatic lesions rapidly release IL - 17 and IFN-γ in response to IL - 23, serving as a key source for maintaining and amplifying local inflammation (12). Moreover, these cells establish a stable tissue-resident state and respond to external stimuli via critical signaling axes such as STAT3 and T-bet, endowing them with rapid effector functions. Additional experiments revealed that such innate lymphoid cells can independently drive inflammation even in the absence of T cells (8), highlighting their autonomous pathogenic potential in psoriasis relapse.

#### 4.1.3 Metabolomics: NK cell metabolic reprogramming and pathological activation

Similar to T cells, NK cell activation is accompanied by profound metabolic reprogramming, including enhanced glucose uptake, upregulated oxidative phosphorylation, and activation of the mTOR pathway. Such metabolic rewiring not only shapes their effector functions but also determines their persistence and amplification capacity within psoriatic lesions (31). Studies have shown that local lactate accumulation and hypoxic microenvironments in psoriatic lesions skew NK cells toward an IL - 17-producing phenotype, thereby enhancing their pathogenicity (31).

Furthermore, in chronic inflammatory settings, NK cells display increased reliance on glycolysis, with the secretion of IL - 17 and GM-CSF tightly associated with glycolytic metabolite accumulation, reflecting their dynamic and plastic metabolic states. Recent evidence also demonstrates that oxidative stress and mitochondrial dysfunction are critical drivers of pathological NK cell activation (32). Persistent ROS burden in chronic psoriatic inflammation activates NF-κB and STAT3 pathways, promoting continuous pro-inflammatory cytokine release; concurrently, elevated mTORC1 activity and impaired AMPK signaling serve as pivotal bridges between metabolism and effector function (33). Targeting these metabolic pathways (e.g., with AMPK agonists or mTORC1 inhibitors) may partially reverse the pro-inflammatory phenotype of NK cells, providing novel strategies for immunometabolic interventions in psoriasis.

#### 4.1.4 Targeting NK cells in clinical strategies

Current treatments for psoriasis primarily focus on T cell-related pathways. However, with the deepening understanding of innate immune mechanisms, NK cells have gradually emerged as one of the potential therapeutic targets. Studies have shown that dimethyl fumarate (DMF) and its metabolite monomethyl fumarate (MMF) can significantly regulate the number and function of NK cells. After DMF treatment, the proportion of CD56bright NK cells in peripheral blood significantly increases. This subset, mainly characterized by immune regulatory functions, can clear activated autoreactive T cells through the release of perforin, granzyme B, and granzyme K. Further *in vitro* experiments have found that both DMF and MMF can enhance the degranulation capacity of NK cells and increase their cytotoxic activity against activated T cells, potentially playing a role in limiting adaptive immune-mediated inflammation (34). This mechanism suggests that the anti-inflammatory effects of DMF-class drugs are not limited to T/B cell regulation but may also be achieved by enhancing the immune suppressive functions of NK cells.

Further research has shown that IL - 23 plays a key role in maintaining the pathogenic phenotype of NK-like cells in psoriatic tissues. IL - 23 not only promotes the secretion of IL - 17A and GM-CSF by NK cells but also helps stabilize the inflammatory microenvironment (35). In psoriasis models, blocking the IL - 23 signaling pathway not only reduces T cell-mediated inflammatory responses but also significantly inhibits the expression of pro-inflammatory cytokines by NK-like cells, demonstrating a dual immune regulatory effect on “non-classical effector cells.” Moreover, GM-CSF, identified as a key downstream molecule of NK cells, plays an important role in inducing keratinocyte stress responses and immune cell infiltration, providing a basis for developing new therapeutic strategies targeting GM-CSF.

In summary, future efforts should build on existing treatment systems to further construct multi-target immune intervention strategies, incorporating innate immune-related pathways such as IL - 23, GM-CSF, and TSLP into an integrated targeting system. Such strategies are expected to enhance the fine-tuning of complex immune networks, especially for refractory psoriasis patients with poor traditional treatment responses, frequent disease recurrence, or persistent tissue inflammation. They hold significant translational application prospects.

### 4.2 Research trends

#### 4.2.1 Exploration of immune comorbidities

Psoriasis is not only a localized inflammatory skin disease but also a phenotype of systemic immune dysregulation, with significantly increased risks of comorbidities with various immune-mediated diseases. Recent studies have found that common immune comorbidities in psoriasis patients include ulcerative colitis, ankylosing spondylitis, rheumatoid arthritis, Hashimoto’s thyroiditis, etc. Shared genetic susceptibility loci and immune regulatory pathways may underlie these associations (36). Particularly, the IL - 23/IL-17 axis, TSLP, and IFN signaling collectively participate in the initiation and maintenance of chronic inflammation in different organ systems.

Research also indicates that NK cells may serve as “common effector cells” in multiple comorbid diseases, exhibiting tissue-specific inflammatory profiles in different tissues. In models of psoriatic arthritis and inflammatory bowel disease, NK cells show high expression of IL - 17A and IFN-γ, suggesting their role as a key immune hub connecting the skin-joint-gut axis. This understanding prompts a shift from a “skin-centric” model to a “systemic immune imbalance” model, emphasizing the integrative role of innate lymphoid cells, including NK cells, in systemic disease spectra (37). Therefore, identifying the functional states and migration patterns of NK cells in multi-system immune comorbidities will not only help reveal the systemic inflammatory mechanisms of psoriasis but also provide a theoretical basis for combined intervention strategies, promoting a transition from organ-specific treatments to multi-system immune modulation.

#### 4.2.2 Stage-specific intervention strategies

The progression of psoriasis encompasses distinct stages—including initial activation, chronic maintenance, and recurrence susceptibility—each characterized by differences in immune cell subsets, activation states, and inflammatory mechanisms (38). Therefore, designing targeted and precisely timed, stage-specific intervention strategies is essential for enhancing therapeutic efficacy and delaying disease relapse.

In the acute phase, natural killer (NK) cells upregulate activating receptors such as NKG2D and CD69, leading to robust secretion of IFN-γ and TNF-α. These cytokines stimulate keratinocytes to produce chemokines and act synergistically with T cells to amplify the inflammatory response (39). Therapeutic interventions at this stage should prioritize the blockade of NK–T cell interaction pathways to rapidly mitigate the inflammatory cascade.

During the chronic phase, the therapeutic focus shifts toward maintaining local immune homeostasis. Prolonged exposure of NK cells to bacterial metabolites and signals from epidermal damage may induce a sub-exhausted or pathologically activated state, thereby sustaining a low-grade chronic inflammatory milieu. Intervention strategies in this phase should aim to modulate cellular metabolism and reprogram NK cell function to stabilize the immune microenvironment.

In the remission phase, tissue-resident NK cells (CD49a^+^CD103^+^) exhibiting features of “trained immunity” can be rapidly reactivated upon re-exposure to stimuli, triggering disease recurrence. Recent studies have demonstrated that bioactive compounds from natural medicines—such as paeoniflorin—can ameliorate psoriatic pathology by suppressing NK cell activation pathways and alleviating oxidative stress in skin tissues, offering a promising approach for remission-phase intervention (40).

In summary, implementing precisely targeted immunomodulatory strategies tailored to each immunological stage may disrupt the “relapse–recurrence” cycle, prolong remission duration, and ultimately improve long-term disease control in psoriasis.

## 5 Conclusion

The role of NK cells in psoriasis has emerged as a cutting-edge focus in immunological research, with ongoing in-depth exploration of their immune functions, metabolic states, and therapeutic potential. Current research is concentrated on three main hotspots: First, tissue-resident NK cells have been confirmed to possess “trained immunity” characteristics with significant pathogenic potential in psoriasis recurrence, particularly their rapid reactivation capability during disease remission. Second, metabolic reprogramming during NK cell activation is decisive for their pro-inflammatory functions, with enhanced glycolysis, hypoxia adaptation, and ROS signaling collectively shaping their pathological state. Third, clinical strategies targeting NK cells are gradually taking shape, with drugs such as DMF and IL - 23 inhibitors demonstrating therapeutic potential through direct or indirect modulation of NK cells, offering new pathways for controlling inflammation beyond T cell - dominated mechanisms.

In terms of future development trends, research is progressively expanding from a focus on localized inflammation to a systemic perspective of immune comorbidities. NK cells may serve as a crucial link between psoriasis and other autoimmune diseases. Meanwhile, the concept of devising stage-specific intervention strategies based on the disease course of psoriasis is emerging, emphasizing precise modulation of NK cell activity at different stages to potentially break the cycle of recurrent disease episodes and improve long-term therapeutic outcomes. Overall, a systematic dissection of the functional spectrum and dynamic features of NK cells in psoriasis will provide a solid foundation for disease mechanism studies and personalized treatments, propelling the field into a new era of more precise and efficient immune interventions.

## Data Availability

The original contributions presented in the study are included in the article/supplementary material. Further inquiries can be directed to the corresponding author.
